# Multiple Non-Species-Specific Pathogens Possibly Triggered the Mass Mortality in *Pinna nobilis*

**DOI:** 10.3390/life10100238

**Published:** 2020-10-13

**Authors:** Fabio Scarpa, Daria Sanna, Ilenia Azzena, Davide Mugetti, Francesco Cerruti, Sepideh Hosseini, Piero Cossu, Stefania Pinna, Daniele Grech, David Cabana, Viviana Pasquini, Giuseppe Esposito, Nicoletta Cadoni, Fabrizio Atzori, Elisabetta Antuofermo, Piero Addis, Leonardo Antonio Sechi, Marino Prearo, Simone Peletto, Marianna A. Mossa, Tiziana Saba, Vittorio Gazale, Marco Casu

**Affiliations:** 1 Dipartimento di Medicina Veterinaria, Università degli Studi di Sassari, Via Vienna 2, 07100 Sassari, Italy; fscarpa@uniss.it (F.S.); iazzena@uniss.it (I.A.); picossu@uniss.it (P.C.); gsesposito@uniss.it (G.E.); eantuofermo@uniss.it (E.A.); marcasu@uniss.it (M.C.); 2 Dipartimento di Scienze Biomediche, Università degli Studi di Sassari, Viale San Pietro 43/B, 07100 Sassari, Italy; s.hosseiniporgham@studenti.uniss.it (S.H.); sechila@uniss.it (L.A.S.); 3 Istituto Zooprofilattico Sperimentale del Piemonte, Liguria e Valle d’Aosta, Via Bologna 148, 10154 Torino, Italy; davide.mugetti@izsto.it (D.M.); francesco.cerutti@izsto.it (F.C.); marino.prearo@izsto.it (M.P.); simone.peletto@izsto.it (S.P.); 4 IMC-International Marine Centre, Loc. Sa Mardini, Torregrande, 09170 Oristano, Italy; s.pinna@fondazioneimc.it (S.P.); d.grech@fondazioneimc.it (D.G.); d.cabana@fondazioneimc.it (D.C.); 5 Dipartimento di Scienze della Vita e dell’Ambiente, Università di Cagliari, Via Fiorelli 1, 09126 Cagliari, Italy; vivi.pasquini@yahoo.it (V.P.); addisp@unica.it (P.A.); 6 Area Marina Protetta Capo Carbonara, Comune di Villasimius, Via Roma 60, 09049 Villasimius, Italy; nicolettacadoni@gmail.com (N.C.); direzione@ampcapocarbonara.it (F.A.); 7 Regiona autonoma della Sardegna, Via Roma 80, 09123 Cagliari, Italy; mmossa@regione.sardegna.it (M.A.M.); tsaba@regione.sardegna.it (T.S.); 8 Area Marina Protetta “Isola dell’Asinara”, 07046 Porto Torres, Italy; gazale@asinara.org

**Keywords:** fan mussel, *Mycobacterium* sp., *Haplosporidium pinnae*, *Rhodococcus erythropolis*, multifactorial disease, Sardinia, heterologous host species, sentinel species, *Pinna rudis*

## Abstract

The fan mussel, *Pinna nobilis*, represents the largest bivalve endemic to the Mediterranean Sea. Since 2016, dramatic mass mortality of this species has been observed in several areas. The first surveys suggested that *Haplosporidium pinnae* (currently considered species-specific) was the main etiological agent, but recent studies have indicated that a multifactorial disease may be responsible for this phenomenon. In this study, we performed molecular diagnostic analyses on *P. nobilis*, *P. rudis*, and bivalve heterologous host species from the island of Sardinia to shed further light on the pathogens involved in the mass mortality. The results support the occurrence of a multifactorial disease and that *Mycobacterium* spp. and *H. pinnae* are not necessarily associated with the illness. Indeed, our analyses revealed that *H. pinnae* is not species-specific for *P. nobilis*, as it was present in other bivalves at least three years before the mass mortality began, and species of *Mycobacterium* were also found in healthy individuals of *P. nobilis* and *P. rudis*. We also detected the species *Rhodococcus erythropolis*, representing the first report in fan mussels of a bacterium other than *Mycobacterium* spp. and *Vibrio* spp. These results depict a complicated scenario, further demonstrating how the *P. nobilis* mass mortality event is far from being fully understood.

## 1. Introduction

The fan mussel (or noble pen shell), *Pinna nobilis* Linnaeus, 1758 (Bivalvia: Pinnidae), is one of the best-known marine invertebrates, representing an endemic flagship species for the Mediterranean Sea. The fan mussel is one of the largest bivalves in the world and the largest in the Mediterranean, reaching up to 120 cm in height [1], and it is one of the most long-lived, living up to 50 years in favorable conditions [2]. *Pinna nobilis* lives at depths between 0.5 and 60 m on soft bottoms overgrown by seagrass meadows [3] and occasionally on the bare sandy substrate and maerl beds [4]. The length of the larval life cycle, as well as factors influencing survival rates and behavior, remain poorly understood [5]. In general, the larval duration for the genus *Pinna* has been estimated to be a maximum of 10 days [4,6]. After the veliger stage in which the larvae drift in the water column [7], juveniles settle in the sediment, anchoring via a byssus [6].

In the 1980s, there were large decreases in most populations of *P. nobilis* due to various anthropogenic activities, such as recreational and commercial fishing, ornamental harvesting (the byssus was used by artisanal manufactures of the so-called ‘sea silk’ [8]), and accidental killing by anchoring and trawl nets [9]. To address the decreased populations, *P. nobilis* was included in a protection regime under the Annex II of the Barcelona Convention (SPA/BD Protocol 1995) and Annex IV of the EU Habitats Directive (European Council Directive 92/43/EEC). More recently, in Italy, the Legislative Decree 190/2010, Art. 11 for the Marine Strategy Monitoring Program included the fan mussel among the Mediterranean species worthy of particular attention. The application of these conservation measures led to the recovery of the populations of *P. nobilis* throughout the entire Mediterranean basin. The results from one of the most recent molecular surveys [10] also supported the good health status of this species, without evidence of genetic fragmentation within and among the main Mediterranean basins [10,11]. 

Unfortunately, since 2016, dramatic mass mortality of the fan mussel populations has been observed in several Mediterranean areas, from the coasts of Spain eastward [12]. To date, epidemiological surveys have indicated different etiological agents as being responsible for the *P. nobilis* mass mortality, with *Haplosporidium pinnae* as the primary agent [13,14,15], followed by *Mycobacterium* sp. [16], *Vibrio* spp. [17,18], and *Perkinsus* spp. [19]. In the first phase of the illness, individuals affected by this unknown disease are characterized by an anomalous slow closure of the valves after external stimulation. In the advanced phases, individuals are no longer able to completely close the valves, becoming highly vulnerable to predation. In the terminal stage, the mantle of the fan mussel detaches from the valves and moves down in the bottom of the shell, after which individuals cannot survive more than a few days (in the absence of predation). 

**Figure 1:**
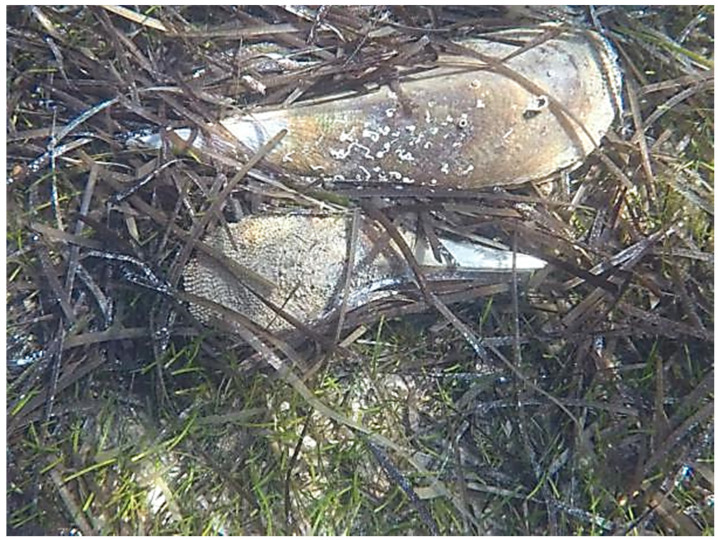
Individuals of *Pinna nobilis* found dead with shell completely detached from the bottom.

In the Western Mediterranean, until a few years ago, Sardinia Island housed a large number of *P. nobilis* populations, which included thousands of individuals (personal observation during sampling collections for previous studies [10,11], mainly located in national parks and marine protected areas distributed along the coastline of the island). However, the Sardinian populations of *P. nobilis* have also been rapidly affected by this mass mortality, which dramatically decimated the island’s populations of fan mussels [16]. In particular, the case of Asinara Island (north-west of Sardinia) presents a brutal picture of the suddenness and severity of the phenomenon along the Sardinian coasts. Indeed, at the beginning of 2018, during a fan mussel census activity in the Asinara Island promoted by the Marine Strategy Monitoring Program, we detected the first signs of decline in *P. nobilis* when the total number of individuals at the sampling sites, considered in the present research, was still over 500 [20]. Then, in July 2018, mortality reached about 50%; in November, almost 90% of individuals were found dead (Figure 1), and their shells were often used as a den for other species. Mass mortality phenomena involving other zoobenthic species (e.g., restricted to bivalves, *Arca noae*, *Spondylus gaederopus*, *Lithophaga lithophaga*, *Ostrea edulis*, *Lima* spp.) have been observed in the past. These events were seen as a consequence of the current climate warming trend [21], which affects more than just filter feeders. However, the situation of *P. nobilis* does not properly fall under such cases and cannot be compared with any other known mortality phenomena to date, at least in the Mediterranean. Indeed, to our knowledge, such a severe event, with very high mortality rates and wide geographical and temporal distributions, has never been reported in Mediterranean zoobenthic marine taxa, making the need to find a solution to stem (or partially stem) this catastrophic scenario even more urgent.

Therefore, in 2019, the government of the Autonomous Region of Sardinia (RAS) promoted an action plan focused on *P. nobilis* populations on the Sardinian coast to evaluate the impact of the massive mortality on its populations using census activities and diagnostic analyses. The aim of this latter task was to improve the knowledge regarding the factors involved in the disease that is leading *P. nobilis* to the brink of local extinction in Sardinia and to shed further light on its mass mortality. Indeed, the island of Sardinia, with its central position in the Western Mediterranean basin, represents a strategic site to perform molecular analyses to enable a better understanding of this phenomenon.

In this context, our research focused on molecular surveys on *P. nobilis* samples collected in Sardinia during 2018 and 2019 to provide a more complete picture of the presence of environmental etiological agents that may be affecting local populations of the species. Based on previous studies [14,16,17], we focused our efforts on identifying protozoans and bacteria. In addition, we selected two Mediterranean bivalve heterologous host species as potential ‘sentinel organisms’ (i.e., *Mytilus galloprovincialis*, collected in 2019, and *Ruditapes decussatus*, collected in 2014) to check, using molecular tools, for the presence of infections by etiological agents in bivalves other than the fan mussel, in both the present and past years. Furthermore, samples from two individuals of *Pinna rudis* were subjected to the same molecular analyses used for *P. nobilis* to verify the possible presence of the etiological agents in a congeneric species.

## 2. Materials and Methods

### 2.1. Sample Collection

**Figure 2:**
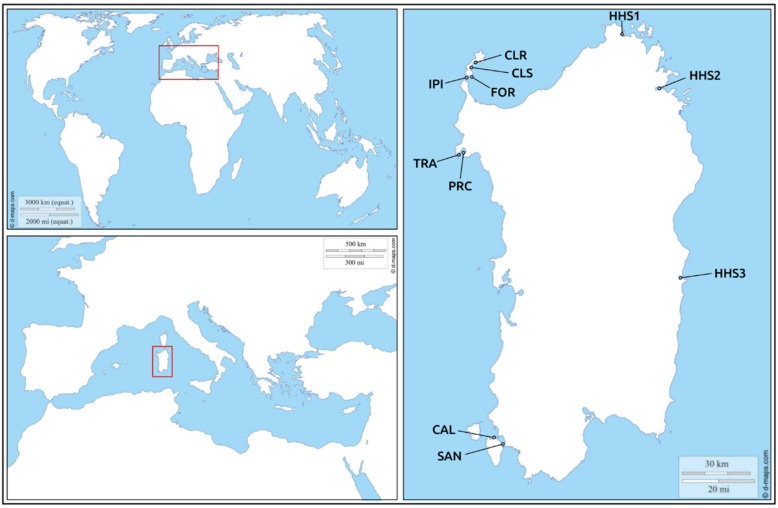
Sampling sites of *Pinna nobilis*, *Pinna rudis*, and heterologous host species (HHS) throughout the coast of Sardinia Island. Sampling site codes are reported in Table 1. In the site IPI, we collected *Pinna nobilis* and *Pinna rudis*. HHS1: *Mytilus galloprovincialis* from Olbia; HHS2 *Ruditapes decussatus* from Porto Pozzo; HHS3: *Ruditapes decussatus* from Tortolì. The maps used are available at the site: https://d-maps.com/index.php?.

Specimens of *P. nobilis* were collected in 2018 (from July to December) and 2019 (from April to December) during the censuses for the Marine Strategy and for the regional action plan, respectively, which involved the entire coastline of the Western Mediterranean island of Sardinia. Because of the severe extent of the mass mortality, after this extensive project, we were able to find only 48 fan mussels on the western coast of the island (see Figure 2 and Table 1 for details on sampling locations and Table S1 in supplemental materials for morphometries). In particular, we collected in situ small fragments of the mantle tissue from 46 of these individuals, which were still alive at the moment of sampling, by using a non-lethal and minimally invasive technique [10] approved by the Italian “Istituto Superiore per la Protezione e la Ricerca Ambientale (ISPRA)” and “Ministero dell’Ambiente e della Tutela del Territorio e del Mare”. Among the 46 live specimens of *P. nobilis* that were collected, 21 exhibited the signs of the disease and 25 did not. During the sampling collection of mantle tissue fragments, animals were classified as apparently sick when the general signs of the disease were found. In addition, tissues from four different organs (i.e., mantle, gills, digestive gland, and adductor muscle) were also collected from the whole body of two individuals from the north-west coast of Sardinia after their death. These two individuals were not classified as sick because during the census activity (occurred a few days before the sampling collection) they showed the lack of any signs of disease. The same non-lethal sampling technique was used to collect mantle samples from two individuals of the congeneric species *P. rudis* from the north-west coast of Sardinia (see Figure 1 and Table 1 for details); these individuals were classified as asymptomatic after the valve closure test. 

Regarding the heterologous host bivalves, we analyzed five individuals of *Mytilus galloprovincialis* (Bivalvia: Mytilidae), collected during 2019 on the north-east coast of Sardinia (Olbia, SS1-40°55′09.5″ N 9°31′07.8″ E), and five individuals of *Ruditapes decussatus* (Bivalvia: Veneridae), collected during 2014 on the central-east coast of Sardinia (two from Porto Pozzo, SS2-41°11′49.9″ N 9°16′30.4″ E, and three from Tortolì, SS3-39°56′26.6″ N 9°40′18.8″ E). For these specimens, DNA was extracted from portions of the mantle and abductor muscle.

### 2.2. Diagnostic Analysis

**Figure 3:**
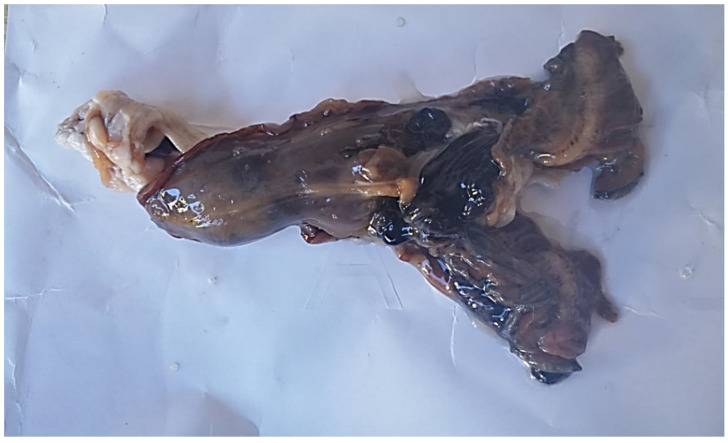
Whole body of the individual of *Pinna nobilis* that was collected immediately after dying (PN19 in Table 1).

The total genomic DNA of bivalves was isolated from a portion of mantle tissue for 46 living individuals. Furthermore, samples from tissues of four different organs (i.e., mantle, gills, digestive gland, and adductor muscle) were collected from the whole body of two individuals of *P. nobilis* after their death (PN1 and PN19 in Table 1). One of these two individuals (PN19) was intact and perfectly preserved: it had been spotted when it was still alive with no signs of disease; therefore, it was monitored daily by scuba divers and sampled immediately after its death to avoid tissue loss by predation and/or bacterial contamination resulting from the *post mortem* deterioration of tissues (see Figure 3). For this individual, molecular analyses were also performed on DNA isolated from hemolymph. 

DNA was extracted from tissue samples using the Macherey-Nagel Nucleo Spin Tissue Kit (MACHEREY-NAGEL GmbH & Co. KG; Neumann Neander Str. 6-8 D-52355 Düren, Germany) following the supplier’s instructions. All specimens were screened for pathogen DNA using standard PCR with primers targeting the protozoan nuclear 18S and bacterial 16S rRNA regions (see [13,16,17] for primer sequences). In addition, the diagnostic PCRs were carried out also on specimens of *M. galloprovincialis* collected in 2019 and specimens of *R. decussatus* collected during the course of 2014 to search for evidence of present and past infections in species other than *P. nobilis* (used here as a potential sentinel species). In these heterologous host species, DNA was extracted from fragments of two organs, the mantle and abductor muscle. All the PCRs were carried out in a total volume of 25 μL. Ten nanograms of total genomic DNA on average (quantified using NanoDrop^TM^ Lite by Thermo Scientific; 81 Wyman Street, Waltham, MA, USA) was combined with 0.6 μM of each primer and one pellet of PuReTaq Ready-To-Go PCR beads (GE Healthcare; 9900 West Innovation Drive, Wauwatosa, WI, USA) containing stabilizers, Bovine Serum Albumin (BSA), deoxynucleotide triphosphates, 2.5 units of PuReTaq DNA polymerase, and reaction buffer. For each bead that was reconstituted to a 25 μL final volume, the concentration of each dNTP was 200 μM and MgCl_2_ was 1.5 mM. PCRs were performed in a GeneAmp PCR System 9700 Thermal Cycler (Applied Biosystems; 81 Wyman Street, Waltham, MA, USA) using the following program: 1 cycle of 4 min at 94 °C, 35 cycles of 30 s at 94 °C, 30 s at 54 °C (for the 18S rRNA gene) or 59 °C (for the 16S rRNA gene), and 30 s at 72 °C. In the end, a post-treatment of 5 min at 72 °C and a final cooling at 4 °C were carried out. Both positive (old samples stored in our laboratory that always produce good PCR products for the primers used in the present study) and negative controls were used to test the effectiveness of the PCR protocols and confirmed the absence of possible inhibitors. In particular, the positive DNA controls we used were samples of *Crassostrea gigas* for the detection of *Vibrio* spp. and *Haplosporidium* spp. and samples of *Ostrea edulis* for the detection of *Mycobacterium* spp. Electrophoresis was carried out on 2% agarose gels that were prepared using 1× TAE buffer (sodium boric acid, pH 8.2) and stained with Gel Red Nucleic Acid Stain (Biotium Inc.; 46117 Landing Parkway, Fremont, CA, USA). PCR products were purified using ExoSAP-IT (USB Corporation; 26111 Miles Rd, Cleveland, OH, USA), and both the forward and reverse strands were sequenced (using the same primers used for PCR) by an external sequencing core service (Macrogen, The Netherlands). 

For diagnostic analyses of protozoans and bacteria, the absence of reaction inhibitors in the solutions of extracted DNA was verified by performing at least two PCRs for each sample with species-specific mitochondrial primers (Cytochrome c Oxidase sub. I) for *P. nobilis*, according to the protocol described by Sanna et al. [10,11].

The pathological diagnostic analysis was also performed on PN19, the dead individual whose body was perfectly preserved after its death (Figure 3). We excluded the second individual found dead (PN1) from this analysis because its body, although it showed no signs of tissue decay, had been partially subjected to predation by muricids, suggesting the possible early onset of the bacterial decomposition process. For PN19, four different organ tissue fragments (i.e., mantle, gills, digestive gland, and adductor muscle) were used as the matrix for the bacteriological examination. Culture tests were performed on Columbia Blood Agar (Liofilchem, Italy), Tryptic Soy Agar (TSA) with 2% NaCl, Marine Agar (MA), and Thiosulfate-Citrate-Bile Salts-Sucrose Agar (TCBS); this last medium was used for the Vibrionaceae screen. First, 100 μL of hemolymph was inoculated on each medium, followed by incubation at 22 ± 2 °C for 72 h. Grown colonies were identified preliminarily using Matrix-assisted laser desorption ionization time of flight (MALDI-TOF) Mass Spectrometry (MS) on a VITEK MS system (bioMérieux, Marcy-l’Étoile, Chemin de l’Orme, France). Together with the bacteriological analyses, specific culture tests were carried out to screen for non-tuberculous mycobacteria. First, 100 μL of hemolymph was inoculated into Middlebrook 7H9 broth (Microbiol, Italy) and incubated at 28 ± 1 °C for two weeks. Then, 10 μL of the broth was taken with a sterile loop and used to inoculate selective media for mycobacteria, as described in detail by Varello et al. [22]. DNA was extracted from the grown colonies by boiling and freeze-thawing and subjected to a mycobacteria-specific PCR targeting a 440 bp portion of the hsp65 gene; the reaction protocols followed those reported by Antuofermo et al. [23].

### 2.3. Statistical Analysis

**Figure 4:**
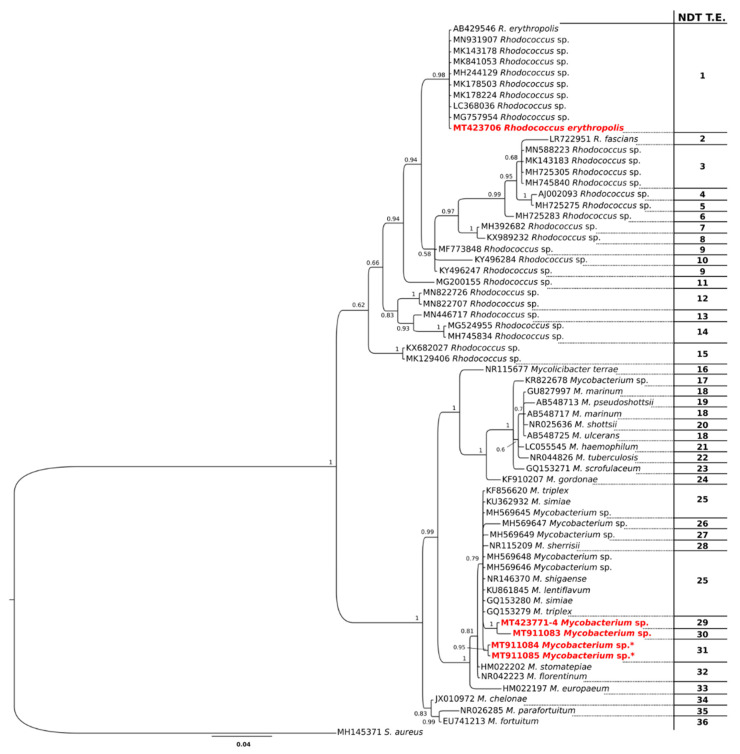
Bayesian phylogenetic tree showing the relationships among bacteria taxa. The column on the right (nucleotide divergence threshold (NDT T.E.) indicates the taxonomic entities found by the NDT method. The sequences obtained in the present study are reported in red font. The asterisk indicates the sequences of bacteria isolated from individuals of *Pinna rudis*.

All the obtained 18S and 16S sequences were used for BLAST searches in the GenBank nucleotide database (NCBI) (https://blast.ncbi.nlm.nih.gov/Blast.cgi?CMD=Web&PAGETYPE=BLASTHome) to search for significant matches (if any) with sequences already reported. The sequences obtained in the present study were then aligned with sequences from conspecifics and congenerics from other localities and hosts downloaded from GenBank (see Figure 4 for accession numbers) using the Q-INS-I algorithm implemented in Mafft 7.187 [24]. 

Regarding the *H. pinnae* 18S marker, we obtained 31 (27 from *P. nobilis* and 4 from the heterologous host species) identical 1205-bp-long sequences (only one sequence was 1216 bp long). 

Regarding the 16S molecular marker, we obtained one *Rhodococcus* sp. sequence from PN19 (*P. nobilis*) and four *Mycobacterium* sp. sequences from the individuals PN19, PN48 (*P. nobilis*), and PR1, PR2 (*P. rudis*). To include the newly obtained sequences in a worldwide context, we first produced a large dataset that contained all of the sequences of the genera *Mycobacterium* and *Rhodococcus* available in GenBank for this bacterial region (i.e., 1,200,135 for *Mycobacterium* spp. and 84,544 for *Rhodococcus* spp.). We then obtained a final version of the dataset after performing several steps of filtering, which took into consideration genetic distances, query cover, identity, and isolation. These filtering steps allowed us to make the dataset as representative and informative as possible for all the sequences considered. The 16S definitive dataset contained 65 sequences with a size of 794 bp, 33 of which belonged to *Mycobacterium* spp. and 31 to *Rhodococcus* spp.; a sequence of *Staphylococcus aureus* was used as an outgroup. Then, tests for the phylogenetic signal were performed using TreePuzzle [25] to verify the dataset reliability [26]. 

Taxonomic assessments and phylogenetic relationships among the investigated taxa were verified using the Bayesian Inference method, which was performed using the software MrBayes 3.2.6 [27], following the method described by Scarpa et al. [28]. The runs were carried out at the Cipres Phylogenetic Portal [29] and checked for the convergence following the procedure described by Ronquist et al. [27] and Gelman and Rubin [30]. The phylogenetic tree was visualized and edited using FigTree 1.4.0 (available at http://tree.bio.ed.ac.uk/software/figtree/). To corroborate the taxonomic assessment obtained according to the phylogenetic species concept, a species delimitation method was also performed using the nucleotide divergence threshold (NDT) using a script [31] written in the R statistical environment, implementing the evolutionary rates of Duchêne et al. [32].

## 3. Results

### 3.1. Protozoa

PCRs were positive for *H. pinnae* in 27 out of the 48 individuals of *P. nobilis* analyzed (56.3%), and this protozoan was present in 71.4% of individuals with signs of disease and in 44.4% of individuals without signs of disease (see Table 1 for details). PCRs were considered negative when two attempts to amplify the same sample did not produce any electrophoretic bands. A large number of infected fan mussels made it possible to test for differences between the occurrence of the signs of disease (the data were collected for all individuals found still living) and the presence of *H. pinnae* in the tissues of individuals using the Pearson’s Chi-squared test (also with Yates’ continuity correction). The individuals with the signs of disease were 21 (15 of which were positive and six negative for *H. pinnae*), while individuals with no signs of disease were 27 (12 of which were positive and 15 negative for *H. pinnae*). The results of this test indicated that the Chi-square statistic amounts to 3.5 (*p* = 0.06) and the Chi-square statistic with Yates’ correction (which is known to be over-cautious for avoiding a type 1 error) amounts to 2.5 (*p* = 0.11). In both cases, taking into account that differences were considered statistically significant at *p* < 0.05, no association between the occurrence of the signs of disease and the presence of the *H. pinnae* was detected. Notably, even if the presence of *H. pinnae* was found in only 56.3% of all the analyzed fan mussels, all these individuals but two died in the meanwhile (see Table 1). Accordingly, the live status of fan mussels (dead vs. alive) was not used as a variable for statistical analyses. It should be pointed out that the lack of environmental data (e.g., pH, salinity, and temperature) might represent a source of bias in this type of analysis. 

All of the 18S rRNA sequences we obtained for *H. pinnae*, both from *P. nobilis* (MT431961–MT431987) and the heterologous host species (MT585819–MT585822), were identical among each other and exactly matched one of the 11 low-variable 18S rRNA sequences previously reported for *H. pinnae* isolated from *P. nobilis* [12,13,14,33,34], thus preventing further phylogenetic inferences.

Regarding the heterologous host organisms, PCRs for *H. pinnae* were positive in three specimens of *R. decussatus* (sampled in 2014) and one specimen of *M. galloprovincialis* (sampled in 2019). These results indicated that these heterologous host species can be considered as sentinel species, as they were hosts for the same pathogens as *P. nobilis*. Accordingly, hereafter, we refer to them as sentinel species. The PCRs for *H. pinnae* were negative for *P. rudis* (see Table 1). 

The DNA samples for which PCRs of Cytochrome c Oxidase sub. I on *P. nobilis*, aimed to verify the lack of inhibitors, did not give bands to the electrophoresis would be considered as contaminated by inhibitors and for this reason unsuitable for these diagnostic tests. Notably, all the DNA samples analyzed in the present study were found PCR inhibitors-free.

### 3.2. Bacteria

In accordance with our Ministerial authorization (approved by ISPRA and the Italian ‘Ministero dell’Ambiente e della Tutela del Territorio e del Mare–Italy’), which allows the collection of only a small piece of the mantle from individuals in a non-lethal manner regardless of their health condition, PN19 was the only sample on which bacteriological examinations were performed because it was found just after it died, perfectly preserved, and without signs of decay (Figure 3). All of the other samples were tested for the presence of bacteria using only the PCR technique. Bacteriological examinations performed on the whole body of the individual PN19 revealed that colonies only formed on Columbia Blood agar. All the isolates were identified as *Rhodococcus erythropolis* using MALDI-TOF MS. Consistently, a 16S rRNA 794-bp-long sequence was obtained (GenBank accession number: MT423706) from a culture obtained from hemolymph that showed 99.87% identity with *Rhodococcus erythropolis* after BLAST analysis. Notably, in the bacteriological examination for the sample PN19, the non-tuberculous mycobacteria screening was negative (no colonies were isolated on selective media).

PCRs of the 16S rRNA fragment performed with DNA extracted from tissues were negative for the presence of bacteria in 46 out of the 48 fan mussel individuals. The only exceptions were the individuals PN19 (from the north-west coast of Sardinia) and PN48 (from the north coast of Sardinia), which were positive for a species of *Mycobacterium*. Interestingly, positive PCR results for bacterial 16S rRNA were also obtained for the *P. rudis* individuals collected along the coasts of Asinara.

As already reported above, also in this case, the DNA samples for which PCRs, aimed to verify the lack of inhibitors, did not give bands to the electrophoresis would be considered as contaminated by inhibitors and for this reason unsuitable for diagnostic tests. Notably, all the DNA samples analyzed in the present study were found PCR inhibitors-free.

BLAST analysis revealed that the 16S rRNA *Mycobacterium* sequence that we obtained for the *P. nobilis* individual PN19 (GenBank accession numbers for identical sequences obtained from the four different organ tissues: MT423771–MT423774) showed a 99.75% identity with *Mycobacterium lentiflavum*, *M. shigaense*, *M. simiae*, *M. triplex*, and several *Mycobacterium* sp., including the uncultured sequences reported by Carella et al. [16]. The sequences obtained for the individual PN48 (GenBank accession number: MT791083) and the two individuals of *P. rudis* (GenBank accession numbers: MT791084–5) showed a 99.62% and 99.87% identity, respectively, with *Mycobacterium lentiflavum*, *M. simiae*, *M. shigaense*, *M. triplex*, and several *Mycobacterium* sp., including the uncultured sequences reported by Carella et al. [16].

No positive PCRs were obtained for *Vibrio* spp. in the present study (see Table 1), possibly because of the methodology we used for the detection. Our *Mycobacterium* and *Rhodococcus* sequences were included in a dataset containing congenerics whose sequences were available in GenBank and selected according to the method described above (see the Materials and Methods section). 

The likelihood-mapping analysis (see Figure S1 in supplementary) indicated that there was a very strong phylogenetic signal, with 4.0% of points in the network-like area, indicating that our data were reliable for phylogenetic and taxonomic inferences [26]. Analyses of the phylogenetic trees (Figure 4) revealed two main clades, with a fully supported dichotomy, representative of the genera *Rhodococcus* and *Mycobacterium*, respectively. The sequences of *Mycobacterium* sp. obtained in the present study were included in a well-supported clade containing *M. lentiflavum*, *M. triplex*, *M. simiae*, *M. sherrisii*, *M. shigaense*, and the *Mycobacterium* sp. sequences isolated from individuals of *P. nobilis* from the Tyrrhenian coastline of Italy [16]. Moreover, the sequence that we obtained for *Rhodococcus* was included in a highly supported clade containing the sequences of *R. erythropolis* and several sequences belonging to the genus without specific taxonomic attribution. 

Notably, the species delimitation analyses, which highlighted a total of 37 taxonomic entities, revealed that the sequences of *Mycobacterium* obtained in the present study belong to three distinct taxonomic entities, which do not include the other sequences of the genus included in the dataset (see Figure 4 for details). On the contrary, the sequence of *Rhodococcus erythropolis* isolated in the present study belongs to the same taxonomic entity that includes the sequences of *R. erythropolis* from GenBank (Figure 4).

## 4. Discussion

Such mass mortality, as dramatic as the one that *P. nobilis* is experiencing, represents an unprecedented phenomenon that has never been recorded also for other species of pinnids [12]. The only quite similar occurrence within Pinnidae was reported for *Atrina pectinata* in Japan during late spring and summer in 2003 and 2004 [35], even if with limited risk for the persistence of the species. In that case, the search for etiological agents provided crucial information to understand the phenomenon. The wide distribution range of *A. pectinata* may explain its good recovery: the extensive spreading of this species into the Indo-Pacific may have helped to prevent an irreversible effect on its conservation status, although the local extinction of some Japanese populations probably caused a general loss of genetic variability. On the contrary, the chance for a quick recovery of *P. nobilis* following the present mass mortality event could be hindered by its narrow range of distribution, and aggregative patterns of populations [36], as well as the semi-enclosed nature of the Mediterranean Sea [37], which allows pathogens to remain within the basin for a long time. The extinction of *P. nobilis* would severely damage Mediterranean biodiversity, and its effect would extend beyond the loss of a single species. Indeed, *P. nobilis* is considered a key species (habitat former) because it provides a hard substrate in areas with soft bottoms, increasing habitat complexity by providing a surface for other benthic species (algae, sponges, bivalves, polychaetes, etc.) [38,39]. In addition, the fan mussel is a host for symbionts such as the crustaceans *Pontonia pinnophylax* and *Nepinnotheres pinnotheres* [40], and it is also predated by other species (e.g., the cephalopod *Octopus vulgaris*), playing a key role in the trophic web [41]. This study sheds further light on the intricate patterns that are involved in the mass mortality of *P. nobilis* in the Western Mediterranean. Even though our analyses were limited to a small number of samples because of the extremely high mortality rates recorded for *P. nobilis* all along the coasts of Sardinia, the new information we obtained will help to bring us closer to fully understanding the causes of this phenomenon. A complete understanding of the causes of this mass mortality is crucial for developing a successful recovery plan for the species, also considering that its high potential for larval dispersal may favor a rapid recolonization across the Mediterranean basins. Indeed, before mass mortality, *P. nobilis* has been shown to exhibit overall genetic uniformity across the Mediterranean, with a slight genetic break at the level of the Siculo-Tunisian Straits [10], such as other Mediterranean mollusks (see, e.g., [42,43,44]).

Protozoans are typically the first putative pathogens investigated in cases of mass mortality involving bivalves. In particular, haplosporidian endoparasites have been the cause of mortality events for many bivalve populations [45], and they have been extensively studied, especially in mollusks of high commercial interest [46,47,48,49,50,51]. Indeed, *H. pinnae* was initially considered as the potential promoter of the mass mortality of *P. nobilis* [7,8] because it was the first pathogen found in large quantities on dead and moribund fan mussel individuals [12,13,14,15] and because it has been considered to be a specific-species protozoan of *P. nobilis*.

Notably, our results indicated that *H. pinnae* is not species-specific for the fan mussel, as was initially hypothesized [13], as it was found in samples of *M. galloprovincialis* and *R. decussatus*, the last of which were collected in 2014. This finding indicates that this protozoan was present in the Mediterranean at least two years before the beginning of the *P. nobilis* mass mortality event (since 2016) [12,13,14,15] and sheds doubts on the hypothesis that *H. pinnae* played a pivotal role in the mass mortality of fan mussels.

Moreover, in accordance with what has already been suggested by Carella et al. [19] (and references therein) for individuals from the northern coast of Spain and by Lattos et al. [33] for Greek populations of the Thermaikos Gulf, our study further supports a hypothesis that is gaining popularity that *P. nobilis* mass mortality is not linked exclusively to infection by the protozoan *H. pinnae* [16,17,18,19,33] (and references therein). In this context, we detected no association between the occurrence of the typical signs of disease responsible for the death of individuals of *P. nobilis* and the presence of *H. pinnae* in their tissues. We found the occurrence of *H. pinnae* in only just over half of the analyzed fan mussels and in just under half of the living individuals that did not show the signs of illness. In particular, among specimens that resulted positive for the presence of *H. pinnae* (n = 27), only 55.6% showed the typical signs associated with the illness, while 44.4% were apparently healthy. However, it should be noted that the tissue analyzed (a 15 mg portion of muscle from the mantle) is not the target tissue of *H. pinnae*, which has been shown to proliferate in digestive glands, and a possible low concentration of DNA in the mantle tissue may have produced some false-negative results [34]. Unfortunately, access to the target organ with a non-invasive approach is not safely possible in alive specimens. Indeed, in the respect of this protected species that is on the brink of extinction, for which sampling of entire individuals when alive are strictly forbidden, and in accordance with our Ministerial authorization (approved by ISPRA and the Italian ‘Ministero dell’Ambiente e della Tutela del Territorio e del Mare’), we received the permission to collect only a small piece of the mantle from alive individuals, in order not to cause lethal damages, which should be avoided particularly in this crucial moment for the survival of *P. nobilis*. 

Nonetheless, it should be noted that the diagnostic PCRs performed for the two specimens found dead (PN1 and PN19) did not return discrepancies among results from the four different organs. Indeed, we found accordance either for positivity (PN1) or negativity (PN19) to *H. pinnae* in each one of the tested tissues (i.e., mantle, gills, digestive gland, and adductor muscle). 

In such a context, future analyses performed by using RT-PCR will allow us to detect even ephemeral concentration of pathogenic DNA when analyzing a small portion of mantle tissue. 

Our findings are also consistent with those obtained in wild [16] and stabled [17] individuals of *P. nobilis*, which indicated that fan mussels showed the typical traits of the disease associated with the mass mortality without the presence of *H. pinnae*. This evidence further demonstrates the lack of a clear connection between this protozoan and the death of *P. nobilis*. Hence, our results, together with those recently reported [19,33], increasingly support a multifaceted scenario that is more complex than expected, in which the occurrence of a multifactorial disease may be the actual cause for the mass mortality event of *P. nobilis*—namely, the disease is not conveyed by a host species-specific pathogen (as was initially concluded regarding *H. pinnae*) but rather by multiple co-occurring pathogens. In this context, additional environmental data would help to corroborate these results or at least clarify if there is also an abiotic component involved in the mass mortality of fan mussels. Unfortunately, these data were not available for each sampling site during the present study, thus preventing to perform these types of analyses.

Regarding the Sardinian area, it is interesting to note that, for two out of three Sardinian individuals sampled in the central-western coast of the island (Oristano), Carella et al. [19] reported the presence of a pathogenic dinoflagellate, *Perkinsus* sp., associated with *Mycobacterium* sp. and *H. pinnae*. In the present study we detected *Rhodococcus erythropolis* (in cultures from hemolymph) associated with *Mycobacterium* sp. in one individual of *P. nobilis* from the northern coast (sample PN19), which represents the first report of a bacterium species in fan mussels other than *Mycobacterium* spp. and *Vibrio* spp. This finding supports the co-occurrence in *P. nobilis* of pathogenic taxa other than those already described [14,16,17]. Moreover, recent results indicate that the mass mortality of *P. nobilis* is likely not just the result of an infection by *Mycobacterium* spp. and *H. pinnae* [14,16]. For this reason, additional studies should be performed to evaluate the roles of the pathogens recently identified, such as *R. erythropolis* and *Perkinsus* sp., in the fan mussel mass mortality. Considering that *R. erythropolis* has been found only in one sample of *P. nobilis*, it is currently difficult to determine its role in the mass mortality event. Indeed, species of *Rhodococcus* are known to have remarkably diverse catabolic abilities [52], but several strains are also able to produce antibiotic type molecules [53]. For instance, Kitagawa and Tamura [54] found antibacterial properties in 80 different strains of *Rhodococcus*, which produced at least three different inhibitory compounds, and 14 of these were taxonomically identified as *R. erythropolis* [54]. These inhibitory molecules show a spectrum of activity against several species of *Rhodococcus* and closely related genera [53]. In this context, the occurrence of *R. erythropolis* could not necessarily be deleterious for the host.

Accordingly, the hypothesis that *R. erythropolis* might provide protection, having a ‘probiotic effect’ on other potential pathogens, cannot be ignored. On the other hand, it must be considered that *R. erythropolis* has been reported to cause illness in Atlantic salmon, *Salmo salar* [55], and in our study, it was found in a dead individual. Thus, based on the current data, we cannot rule out that *R. erythropolis* might be involved, together with other pathogens, in the mass mortality of *P. nobilis*.

Notably, no positive PCRs were obtained for *Vibrio* spp. in the present study, indicating the possible absence of these bacteria among Sardinian fan mussels and agreeing with the trend already reported for Apulian populations [14]. Our phylogenetic analysis showed that the sequences of *Mycobacterium* sp. we obtained are very close to those of species of mycobacteria that are common in marine/aquatic hosts, such as *M. lentiflavum*, *M. triplex*, *M. simiae*, *M. sherrisii*, and *M. shigaense*, and the BLAST analysis revealed high percentages of similarity with several already known species of *Mycobacterium*. The species delimitation results showed that the *Mycobacterium* spp. found in our samples belong to three taxonomic entities that have never been described to date and were reported for the first time in Sardinian samples. This finding is consistent with Carella et al. [19] (and references therein) who also reported the likely occurrence of a new species of mycobacteria infecting *P. nobilis*, which was closely related to *M. triplex* and *M. sherrisii*. This large number of *Mycobacterium* species found to date in the fan mussel, as well as the new possible pathogens that are gradually being discovered, suggests that the overall role of mycobacteria in the mass mortality event, as well as that of *H. pinnae*, should be re-evaluated. This scenario is also supported by the discrepancies in the infection levels between Sardinian individuals analyzed in the present study (2 out of 48 individuals, about 4%) and individuals from the north-west coast of Spain and southern coast of the Tyrrhenian Sea, for which Carella et al. [16,19] reported levels of 50% and slightly less than 100%, respectively.

A species of *Mycobacterium* was also identified by PCR in individuals of *P. rudis* that were still alive (last checked on May 2020) and did not show the signs of the disease at the time of sampling or during periodic checks. This finding suggests the possible presence of several species of *Mycobacterium* in the aquatic environment, although, as in this case, they might not play a pathogenic role. 

## 5. Conclusions

Our results support the occurrence of a multifactorial disease as a possible explanation for the mass mortality of *P. nobilis*, as *Mycobacterium* spp. and *H. pinnae* are not always associated with the illness. Indeed, the lack of *H. pinnae* in many specimens with signs of disease, together with its presence in living individuals with no signs, further suggests that *H. pinnae* cannot be the only cause of this phenomenon. 

In addition, our results from analyses of sentinel species indicate that *H. pinnae* is not species-specific for *P. nobilis*, as it has been present in the Mediterranean in other bivalves at least since 2014, questioning the proposed role of this protozoan in the fan mussel mass mortality event. Future studies should aim to corroborate the molecular results while also using an environmental parameter-based approach. 

Consistently, species of *Mycobacterium* were also found to be present in healthy individuals of *P. nobilis* and *P. rudis*, suggesting that the presence of mycobacteria may be not linked to the causes of the mass mortality, which is likely because these species have been shown to be common etiological agents in the marine environment. In this context, the occurrence of *R. erythropolis* represents the first report of this bacteria species in *P. nobilis*, providing a new candidate for further investigations to clarify its role (if any) in the mass mortality of fan mussels. 

Currently, the causes of this phenomenon are far from being completely understood. Two main factors hinder the clarification of the causes: (i) the lack of a large number of fan mussels to survey and (ii) the impossibility to analyze a proper number of fan mussel entire soft bodies due to the extreme rarity and the levels of total protection to which *P. nobilis* is subjected. In this context, the use of sentinel species may play a pivotal role in identifying the etiological agents involved in the mass mortality, and when combined with more powerful diagnostic methods (such as real-time PCR), they might help overcome the problems related to working on small portions of mantle tissue obtained with a non-lethal sampling method. 

## Figures and Tables

**Table 1:** Sampling localities for the individuals of *Pinna nobilis* (PN) and *Pinna rudis* (PR). For each sample, the presence of the signs of disease (SD), the final status of survival (FS) indicated as alive (A) or dead (D), and detection of potential etiological agents in tissues are indicated. MPA: Marine Protected Areas. Code: sampling sites code.

Sample_ID	Locality	Code	Coordinates	SD	FS	*H. pinnae*	*Mycobacterium* spp.	*Vibrio* spp.
PN1	Sant’Antioco	SAN	39°03′49.2″ N 8°27′47.6″ E	NO	D	YES	NO	NO
PN2				NO	D	YES	NO	NO
PN3				NO	D	NO	NO	NO
PN4				NO	D	NO	NO	NO
PN5	Calasetta-Cussorgia	CAL	39°06′27.7″ N 8°23′54.8″ E	NO	D	YES	NO	NO
PN6				YES	D	YES	NO	NO
PN7				NO	D	NO	NO	NO
PN8				NO	D	NO	NO	NO
PN9				NO	D	NO	NO	NO
PN10				YES	D	NO	NO	NO
PN11				YES	D	NO	NO	NO
PN12				NO	D	NO	NO	NO
PN13				NO	D	NO	NO	NO
PN14				NO	D	NO	NO	NO
PN15				NO	D	NO	NO	NO
PN16				NO	D	NO	NO	NO
PN17				NO	D	YES	NO	NO
PN18	Tramariglio-MPA Capo Caccia Isola Piana	TRA	40°35′23.6″ N 8°10′12.4″ E	NO	D	YES	NO	NO
PN19				NO	D	NO	YES	NO
PN20	Porto Conte-MPA Capo Caccia Isola Piana	PRC	40°35′46.7″ N 8°12′58.1″ E	NO	A	NO	NO	NO
PN21	Cala Reale-MPA Isola dell’Asinara	CLR	41°03′47.6″ N 8°16′59.5″ E	NO	D	NO	NO	NO
PN22				YES	D	YES	NO	NO
PN23				NO	D	YES	NO	NO
PN24				YES	D	YES	NO	NO
PN25				NO	D	YES	NO	NO
PN26				NO	D	YES	NO	NO
PN27				YES	D	YES	NO	NO
PN28				YES	D	YES	NO	NO
PN29				YES	D	YES	NO	NO
PN30				YES	D	YES	NO	NO
PN31	Fornelli-MPA Isola dell’Asinara	FOR	40°59′04.1″ N 8°15′06.3″ E	YES	D	NO	NO	NO
PN32				YES	D	NO	NO	NO
PN33				NO	D	NO	NO	NO
PN34				NO	D	YES	NO	NO
PN35				YES	D	NO	NO	NO
PN36				YES	D	YES	NO	NO
PN37				YES	D	YES	NO	NO
PN38	Cala Scombro di dentro-MPA Isola dell’Asinara	CLS	41°01′43.5″ N 8°14′44.6″ E	YES	D	YES	NO	NO
PN39				YES	D	YES	NO	NO
PN40				NO	D	YES	NO	NO
PN41				YES	D	YES	NO	NO
PN42				YES	D	YES	NO	NO
PN43				YES	D	NO	NO	NO
PN44				NO	D	YES	NO	NO
PN45				NO	D	YES	NO	NO
PN46				YES	D	YES	NO	NO
PN47				YES	D	YES	NO	NO
PN48	Isola Piana-MPA Isola dell’Asinara	IPI	40°58′42.9″ N 8°13′22.6″ E	NO	A	NO	YES	NO
PR1				NO	A	NO	YES	NO
PR2				NO	A	NO	YES	NO

## Supplementary Materials

The following tables and figures are available online at https://www.mdpi.com/2075-1729/10/10/238/s1, Table S1: Shell morphometric measurements for the individuals of *Pinna nobilis* (PN) and *Pinna rudis* (PR) that were analyzed in the present study. Figure S1: Likelihood map depicting the distribution of dots P, where P represents the likelihoods of the three possible unrooted trees for a set of four sequences [25,26]. Central area represents a star-like signal (phylogenetic noise). The value above the triangle represents the percentage of dots in the network-like area. A percentage lower than 20–30% indicates that the dataset is reliable for phylogenetic and taxonomic analyses with a good phylogenetic signal [26].
